# Bacteriophages engineered to display foreign peptides may become short‐circulating phages

**DOI:** 10.1111/1751-7915.13414

**Published:** 2019-04-29

**Authors:** Katarzyna Hodyra‐Stefaniak, Karolina Lahutta, Joanna Majewska, Zuzanna Kaźmierczak, Dorota Lecion, Marek Harhala, Weronika Kęska, Barbara Owczarek, Ewa Jończyk‐Matysiak, Anna Kłopot, Paulina Miernikiewicz, Dominika Kula, Andrzej Górski, Krystyna Dąbrowska

**Affiliations:** ^1^ Institute of Immunology and Experimental Therapy Polish Academy of Sciences Weigla 12 Wrocław Poland

## Abstract

Bacteriophages draw scientific attention in medicine and biotechnology, including phage engineering, widely used to shape biological properties of bacteriophages. We developed engineered T4‐derived bacteriophages presenting seven types of tissue‐homing peptides. We evaluated phage accumulation in targeted tissues, spleen, liver and phage circulation in blood (in mice). Contrary to expectations, accumulation of engineered bacteriophages in targeted organs was not observed, but instead, three engineered phages achieved tissue titres up to 2 orders of magnitude lower than unmodified T4. This correlated with impaired survival of these phages in the circulation. Thus, engineering of T4 phage resulted in the short‐circulating phage phenotype. We found that the complement system inactivated engineered phages significantly more strongly than unmodified T4, while no significant differences in phages’ susceptibility to phagocytosis or immunogenicity were found. The short‐circulating phage phenotype of the engineered phages suggests that natural phages, at least those propagating on commensal bacteria of animals and humans, are naturally optimized to escape rapid neutralization by the immune system. In this way, phages remain active for longer when inside mammalian bodies, thus increasing their chance of propagating on commensal bacteria. The effect of phage engineering on phage pharmacokinetics should be considered in phage design for medical purposes.

## Introduction

Bacteriophages (phages), which are among the most abundant biological entities on earth, are extensively studied as important tools for medicine and biotechnology. Natural phages are applicable for antibacterial therapies that make use of the intrinsic ability of phages to kill bacteria. Phage therapy is considered a hope to help overcome the antibiotic‐resistance crisis that we currently face (Kutter *et al*., 2010; Abedon *et al*., 2011; Pirnay *et al*., 2011; Miedzybrodzki *et al*., 2012; Kazmierczak *et al*., 2014; Gorski *et al*., 2016). Biotechnology, in turn, explores phage potential for diverse modifications. This field has been emerging during recent years (Pires *et al*., 2016). Modified phage virions can be versatile and universal nanocarriers, suitable for the delivery of biologically active elements (Jiang *et al*., 1997; Sathaliyawala *et al*., 2006; Yacoby and Benhar, 2008; Yao *et al*., 2016), as well as biosensors capable of detecting and labelling biological targets (Lee *et al*., 2017; Yue *et al*., 2017). Phage engineering has also been proposed to enhance, expand or target biological activity of therapeutic phages *in vivo* when used as antibacterials (Dabrowska *et al*., 2014b; Gorski *et al*., 2015; Pires *et al*., 2016).

Administration of phages to humans or to animals exposes phages to interactions with the immune system (Gorski *et al*., 2012) that eventually determine phage pharmacokinetics (Hodyra‐Stefaniak *et al*., 2015; Van Belleghem *et al*., 2017). Phage pharmacokinetics, in turn, determines phage therapeutic efficacy and outcomes of the treatment (Payne *et al*., 2000; Levin and Bull, 2004; Cairns *et al*., 2009). Although induction of phage‐specific antibodies is by far the most widely investigated aspect of immune reactions to phages (Uhr *et al*., 1962a,b; Ochs *et al*., 1971; Smith *et al*., 1987; Fogelman *et al*., 2000; Huff *et al*., 2010; Dabrowska *et al*., 2014a; Majewska *et al*., 2015; Lusiak‐Szelachowska *et al*., 2017), it is the innate immune response that plays the key role in phage clearance from animal and human bodies in non‐immunized individuals. Innate immunity is non‐specific and it removes phage particles even when no specific response to bacteriophages has yet developed. Non‐specific removal of phages is executed mainly by the mononuclear phagocytic system (MPS, previously: reticulo‐endothelial system or RES), whereby phages are filtered and inactivated by phagocytosis. The spleen and liver, which are key elements of the MPS, have been demonstrated as major ‘phage traps’ inside bodies (Keller and Engley, 1958; Inchley, 1969; Geier *et al*., 1973; Hodyra‐Stefaniak *et al*., 2015). To some extent, bacteriophages can also be neutralized by the serum complement system in a way similar to inactivation of other viruses (Sulkin *et al*., 1957; Hajek and Mandel, 1966; Dabrowska *et al*., 2014a; Hodyra‐Stefaniak *et al*., 2015). Bacteriophages can also affect the innate part of immune response. In general, phage effect on the immune response seems to be anti‐inflammatory. Phage, that naturally binds bacterial products, may moderate signals from bacterial PAMPs (Pathogen‐Associated Molecular Patterns) and thus decrease inflammation (Miedzybrodzki *et al*., 2008; Gorski *et al*., 2012; Miernikiewicz *et al*., 2016; Zhang *et al*., 2018). Recently, direct anti‐inflammatory phage effect on mammalian cells has been detected by gene expression profiling of peripheral blood monocytes. This effect may further affect outcomes of phage therapeutic application (Van Belleghem *et al*., 2017).

Differences in phage susceptibility to neutralization by the innate immune system may be responsible for differences in phage ability to remain active *in vivo*. This was first reported by Merril *et al*. (Merril *et al*., 1996), who isolated *long‐circulating phages*. Long‐circulating phages are mutants or variants capable of maintaining their antibacterial activity for longer (than parental strains) when they circulate in mammalian blood. Bacteriophages present in the circulation immediately disseminate throughout the body (Dabrowska *et al*., 2005); thus, prolonged circulation indicates maintenance of higher titres in the whole body. Long‐circulating phages have been identified as more efficient in phage therapy of experimental septicaemia (Merril *et al*., 1996; Vitiello *et al*., 2005; Capparelli *et al*., 2006, 2007).

As an alternative to searching for phage mutants able to circulate longer in the system, we have previously proposed phage engineering with small peptides that promote phage accumulation in selected tissues (Gorski *et al*., 2015). It is unclear if the modified phages, by adsorption to selected cell types, would leave the circulation and thus result in a short‐circulating phenotype. Nevertheless, increasing phage concentration at the site of infection may be essential for achieving the ‘inundation threshold’, which is the minimum phage density that can prevent a bacterial infection from progressing (Abedon, 2011). In localized infections, this might greatly increase the success of treatment. The targeting (homing) peptides can be presented on the phage surface by phage display technology (Gorski *et al*., 2015). The idea relates to the fundamental studies of Ruoslahti, who identified homing peptides for brain and kidney (Pasqualini and Ruoslahti, 1996). Peptides facilitating delivery and homing to many tissues were further identified by the use of phage display libraries (Arap *et al*., 2002a,b; Duerr *et al*., 2004; Kang *et al*., 2008; Giordano *et al*., 2009; Budynek *et al*., 2010; Li *et al*., 2011, 2012; Teesalu *et al*., 2013), thus demonstrating broad potential to target drugs to selected organs, potentially improving outcomes of many kinds of treatment (Ruoslahti, 2012).

Here, we report a study of seven T4‐derived phages, each presenting targeting peptides on the head surface. Phage virions were engineered by phage display as previously described (Oslizlo *et al*., 2011; Ceglarek *et al*., 2013). They presented peptides targeting the lung (2 peptides), prostate (2 peptides) or brain (1 peptide), or facilitating translocation from the intestine lumen to the circulation (2 peptides). All seven types of engineered phages were investigated *in vivo* for their pharmacokinetics and compared to non‐modified T4 phage. Phage titres were tested in blood and in selected organs including the spleen and liver. To understand individual pharmacokinetics of engineered phages, immune responses elicited by the engineered phages were identified in terms of both the innate immune response (phagocytosis, serum complement activity) and the adaptive immune response (antibodies).

## Results

### Circulation of engineered bacteriophages in targeted tissues, spleen, liver and blood

**Figure 1:**
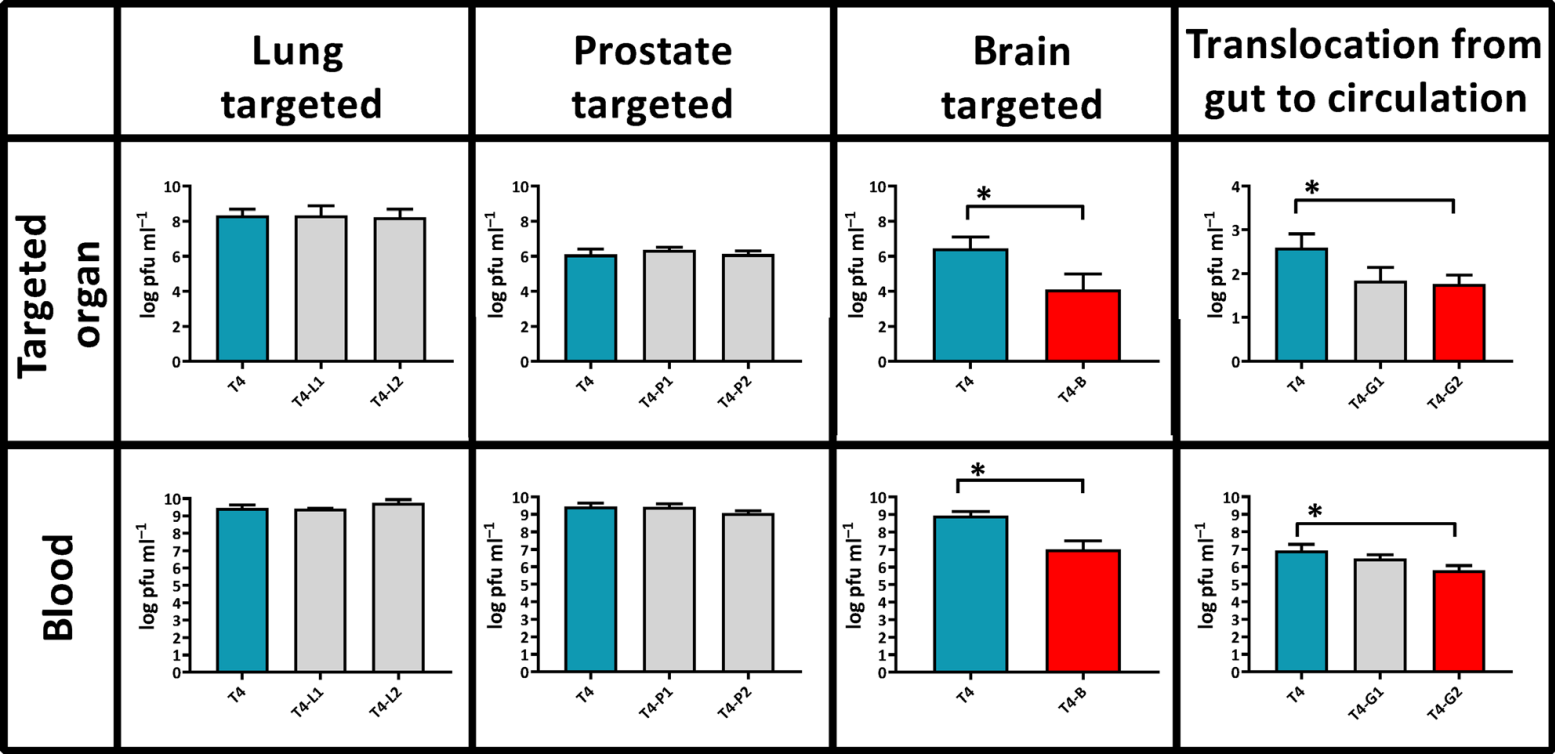
Phage titres in targeted organs and in blood of mice after administration of T4 phage presenting peptides targeting lungs (T4‐L1 and T4‐L2), prostate (T4‐P1 and T4‐P2), brain (T4‐B) and facilitating translocation from gut to circulation (T4‐G1 and T4‐G2). Targeted organs: Mice (*N* = 5 to 6) were injected intravenously with a phage dose of 5 × 10^9^ pfu per mouse (each phage) and phage titre was measured in tissues 2 h later (T4‐L1, T4‐L2, T4‐P1, T4‐P2, T4‐B) or phage was added to drinking water at 5 × 10^10^ pfu per ml and phage titre was measured 10 h later in blood (T4‐G1 and T4‐G2). In each case, non‐modified T4 phage (blue bars) was used as a control. Log_10_ of mean phage concentrations in selected organs (pfu per gram of tissue or per ml of blood) is presented (bars) with standard deviation (whiskers). Differences statistically significant in comparison to control phage are indicated with asterisks and red colour of a relevant bar (Mann–Whitney *U*‐test, *P* < 0.05). Blood: in all cases, mice (*N* = 5 to 6) were injected intravenously with tested phages 1 × 10^9^–1 × 10^10^ pfu per mouse to compare phage circulation in blood, phage titre in blood was measured 2 h later. In each case non‐modified T4 phage (T4, 1 × 10^10^ pfu per mouse) was used as a control. Log_10_ of mean phage concentrations is presented (bars) with standard deviation (whiskers). Differences statistically significant in comparison to control phage are indicated with asterisks and a red bar (Mann–Whitney *U*‐test, *P* < 0.05).

**Figure 2:**
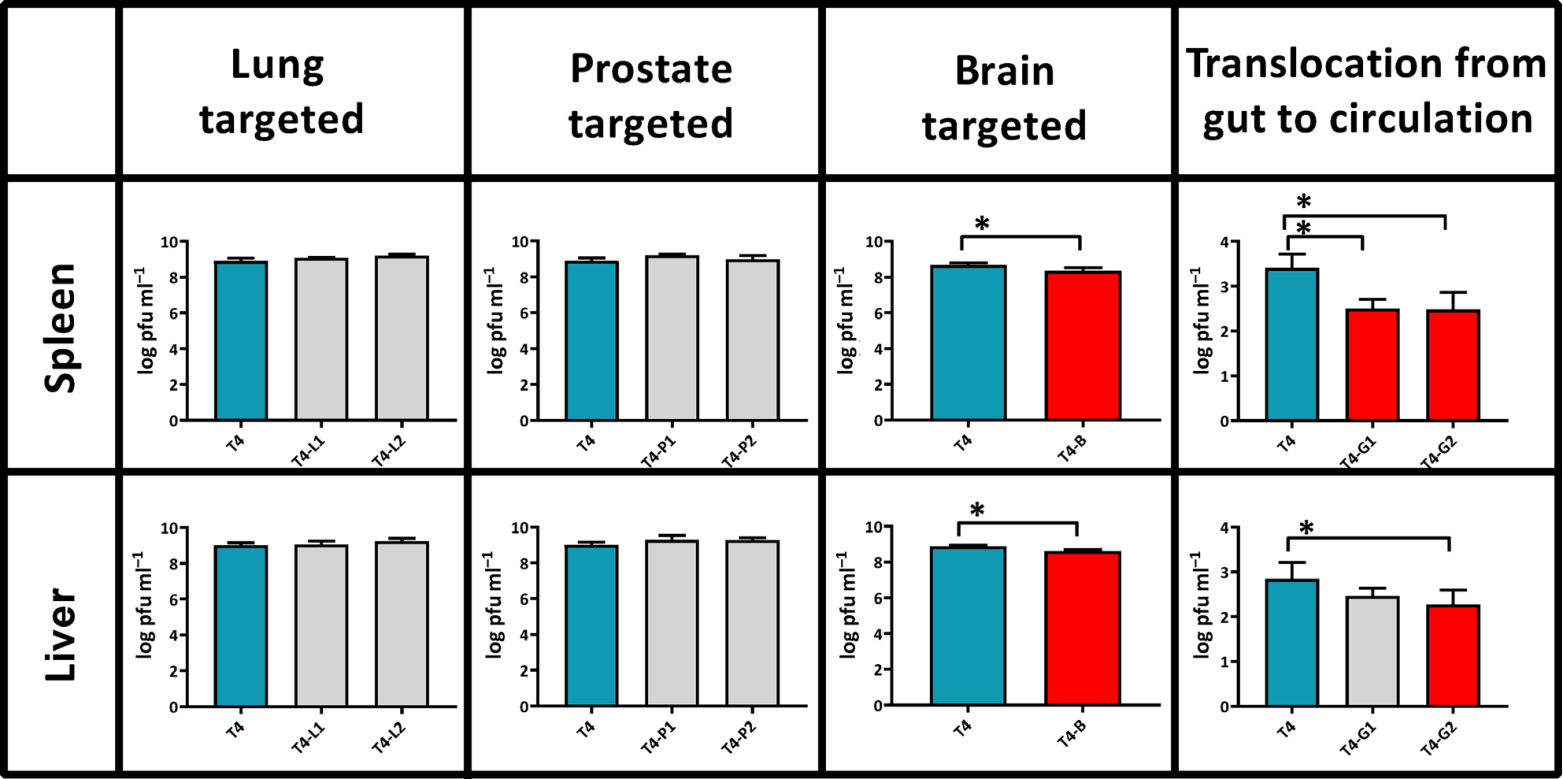
Phage titres in murine spleen and liver after administration of T4 phage presenting peptides targeting lungs (T4‐L1 and T4‐L2), prostate (T4‐P1 and T4‐P2), brain (T4‐B) and facilitating translocation from gut to circulation (T4‐G1 and T4‐G2). Mice (*N* = 5 to 6) were injected intraperitoneally with a phage dose of 5 × 10^9^ pfu per mouse (each phage) and phage titre was measured in tissues 2 h later (T4‐L1, T4‐L2, T4‐P1, T4‐P2, T4‐B) or phage was added to drinking water at 5 × 10^10^ pfu per ml and phage titre was measured 10 h later in blood (T4‐G1 and T4‐G2). In each case, non‐modified T4 phage (blue bars) was used as a control. Log_10_ of mean phage concentrations in selected organs (pfu per gram of tissue or per ml of blood) is presented (bars) with standard deviation (whiskers). Differences statistically significant in comparison to control phage are indicated with asterisks and red colour of a relevant bar (Mann–Whitney *U*‐test, *P* < 0.05).

The following seven types of engineered bacteriophage T4 were constructed; these phages displayed peptides targeting the lungs (T4‐L1 and T4‐L2), the prostate (T4‐P1 and T4‐P2), the brain (T4‐B), and facilitating translocation from the gut lumen to the circulation (T4‐G1 and T4‐G2) (for sequences and references see Table 1 in the Material and Methods section). All engineered phages presented the peptides as N‐terminal fusions to surface protein Hoc. We tested by anti‐Hoc antibody reaction relative saturation of phage particles with Hoc fusions (Fig. S1). We confirmed that Hoc fusions were present on all types of engineered phages. The ability of displayed peptides to target selected cells was confirmed *in vitro* in representative phages; these were demonstrated to bind (T4‐P1, T4‐B) or to translocate across targeted cells (Fig. S2). Investigated bacteriophages were injected i.v. into mice (T4‐L1, T4‐L2, T4‐P1, T4‐P2, T4‐B) or added to drinking water (T4‐G1, T4‐G2). Unmodified T4 phage served as a control in each case and it was applied by identical route and schedule as engineered phages. Eventually, phages disseminated in the whole body, since active phages were detected in all targeted organs as well as in the spleen and liver (Figs 1 and 2). However, expected accumulation of engineered phages in targeted organs was not observed in any case (Fig. 1). We did not observe any cross‐reactivity between types of modified phages (data not shown). Further, phages T4‐B and T4‐G2 achieved approximately 2 orders of magnitude lower titres in targeted organs than the parental strain (Fig. 1), which was opposite to expected outcomes of phage modifications. Concordant results were observed longer after the administration (up to 24 hours after administration) (data not shown).

**Table 1:** Targeting peptides presented on T4 phage

Targeted organ	Designation of phage displaying this peptide	Sequence of targeting peptide	Reference
Lungs	T4‐L1	CGFECVRQCPERC	Rajotte and Ruoslahti (1999)
Lungs	T4‐L2	CGSPGWVRC	Giordano *et al*. (2008)
Brain	T4‐B	TGNYKALHPHNG	Li *et al*. (2011)
Prostate	T4‐P1	RRAGGS	Arap *et al*. (2002b)
Prostate	T4‐P2	SMSIARL	Arap *et al*. (2002a)
Translocation from gut lumen to circulation	T4‐G1	YPRLLTP	Duerr *et al*. (2004)
Translocation from gut lumen to circulation	T4‐G2	CSKSSDYQC	Kang *et al*. (2008)

Intravenous (i.v.) administration of modified bacteriophages was used to assess their ability to survive in the circulation. All investigated bacteriophages were administered i.v. to mice, and unmodified T4 phage served as the control. Two hours after administration, phage titre was determined in blood. No differences were observed between the blood titre of control T4 phage and modified phages T4‐L1, T4‐L2, T4‐P1, T4‐P2 and T4‐G1. However, blood titres of T4‐B and T4‐G2 were from 1 to 2 orders of magnitude lower than those of the unmodified control (*P* = 0.04164 and *P* = 0.007382, respectively) (Fig. 1). Consistent results were observed longer up to 24 h after administration (data not shown). Notably, concentrations of T4‐B and T4‐G2 bacteriophages in the spleen and liver were also significantly lower than concentrations of the unmodified T4 phage (Fig. 2). Lower titres of T4‐B and T4‐G2 in all investigated tissues suggested that those engineered phages had a generally weaker ability to survive in living animal bodies.

### Interactions of engineered bacteriophages with innate immunity

Phage ability to survive in the circulation may depend on phage susceptibility to neutralization by the innate immune system (Merril *et al*., 1996; Hodyra‐Stefaniak *et al*., 2015). Thus, we investigated *ex vivo* interactions of the phages T4‐B, T4‐G1, T4‐G2 and unmodified T4 (control) with two major parts of the innate immunity response: complement system and phagocytes. Phages were incubated with blood sera as the source of complement, and with isolated phagocytic cells which were polymorphonuclear cells (PMNs) or peripheral blood mononuclear cells (PBMCs). Human blood was used in this part of the study to make the observations more useful for therapeutic and other medical solutions in humans.

**Figure 3:**
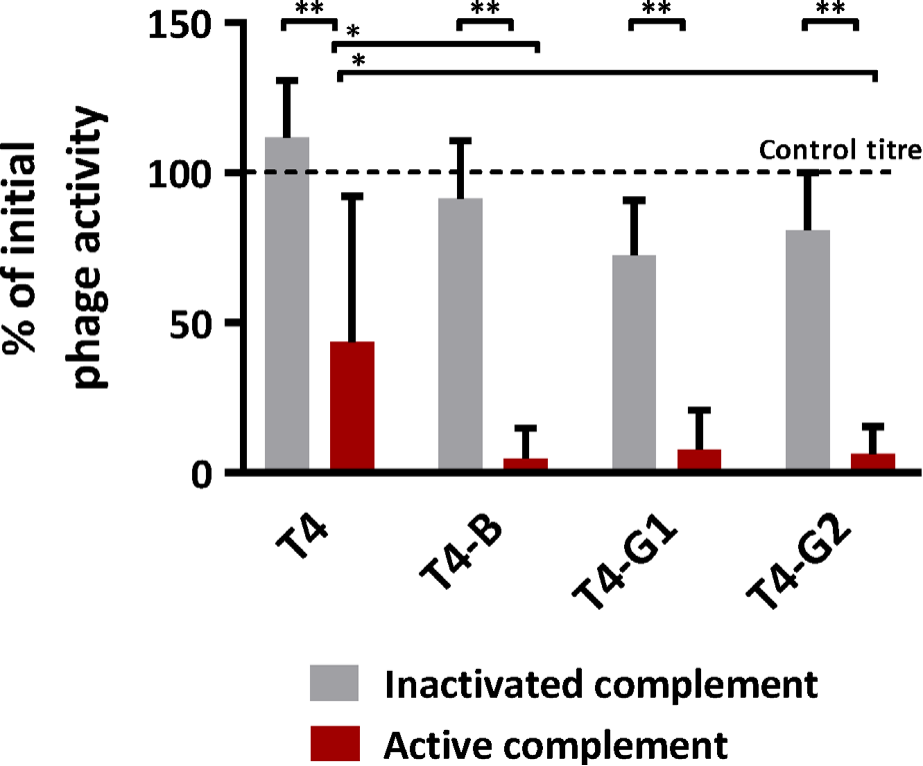
Effect of human complement system *ex vivo* on non‐modified bacteriophage T4 and on T4 presenting peptides targeting brain (T4‐B), and facilitating translocation from gut to circulation (T4‐G1 and T4‐G2). Blood samples from six healthy human volunteers were use. Individuals defective for the serum complement activity were excluded from the study. Serum was isolated from blood samples and incubated 1:1 with phage preparations (10^7^ pfu ml^−1^) for 1 h at 37°C, either active (red bars) or after heat inactivation for 1.5 h at 56°C (grey bars). After incubation, phage activity was tested by the double‐layer plate method. Phage titre was compared to control titre without incubation with serum (initial phage activity) and presented as % of the control. **Statistically significant *P* < 0.001, *statistically significant *P* < 0.05.

**Figure 4:**
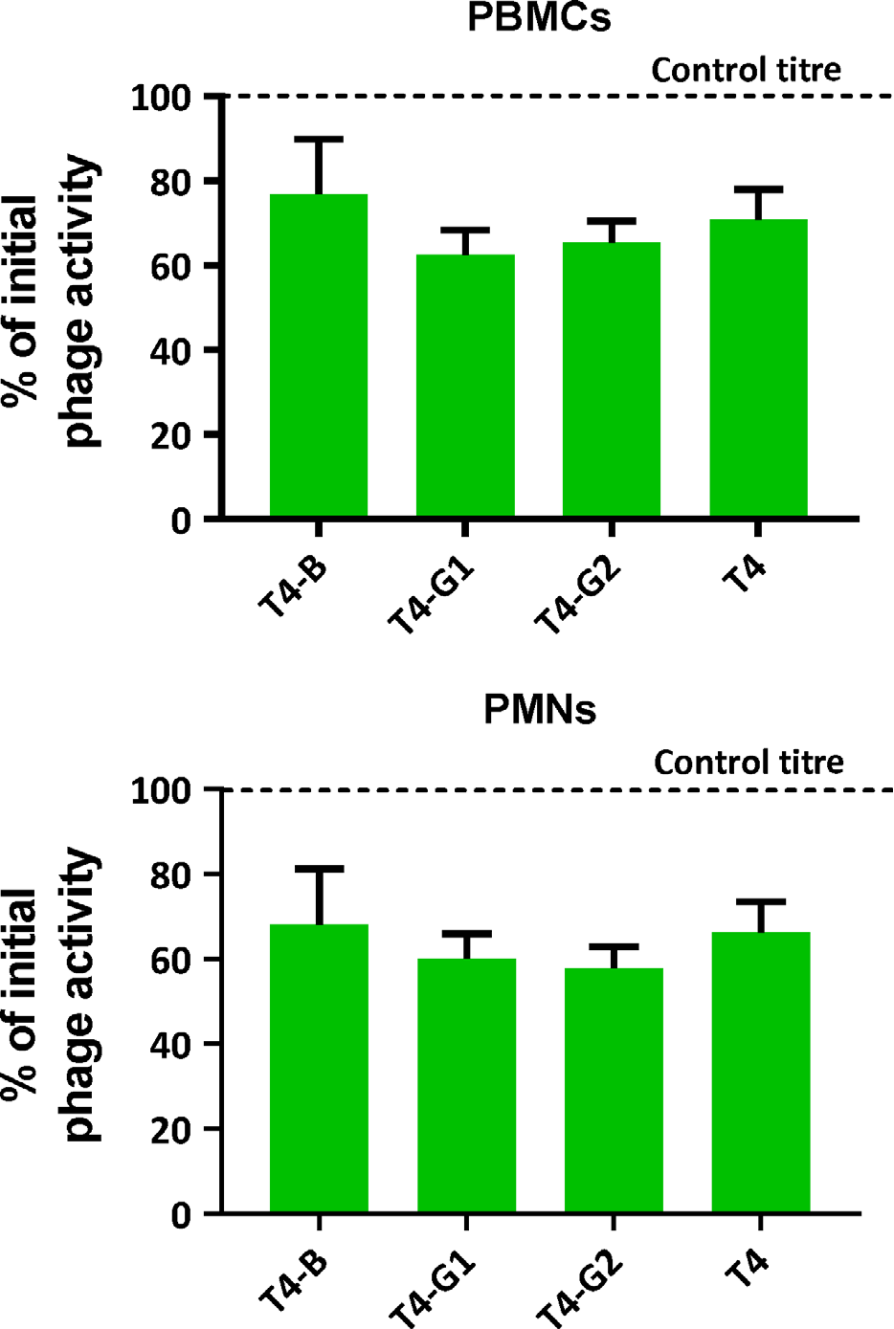
Effect of human phagocytes *ex vivo* on non‐modified bacteriophage T4 and on T4 presenting peptides targeting brain (T4‐B), and facilitating translocation from gut to circulation (T4‐G1 and T4‐G2). Polymorphonuclear neutrophils (PMNs) and mononuclear blood cells (PBMCs) were used in a final density of 10^6^ cells ml^−1^. Phages T4‐B1, T4‐G1, T4‐G2 and T4 as the control were added to a final count of 10^5^/ml (volume: 1 ml) and incubated for 1.5 h at 37°C, viable phage titre detected after incubation was determined by RTD in the culture supernatant, results were presented as the per cent of initial phage titre (control titre).

Exposure of phages to the complement system significantly decreased phage activity: phage titre remaining after incubation with active sera ranged from 4.7% (T4‐B) to 43.7% (T4) of initial phage activity, while it was not decreased in the same phages incubated with inactivated sera (*P* < 0.001) (Fig. 3). Unmodified T4 phage was less susceptible to neutralization by the complement system than phages displaying targeting peptides. This difference was significant when compared to T4‐B and T4‐G2 (*P* = 0.007 and *P* = 0.0485, respectively) (Fig. 3). Incubation of phages with phagocytes, in turn, did not result in significant differences between titres of remaining engineered bacteriophages and control phage T4; the overall decrease of phage titre ranged from 20 to 40% of the initial phage titre (control) (Fig. 4). Thus, individual phage susceptibility to phagocytosis by immune cells does not seem to contribute to differences between engineered and non‐engineered bacteriophages in their ability to survive in circulation *in vivo*, while the complement system plays an important role in inactivation of bacteriophages, having a stronger effect on the engineered ones.

### Induction of antibodies by engineered bacteriophages

**Figure 5:**
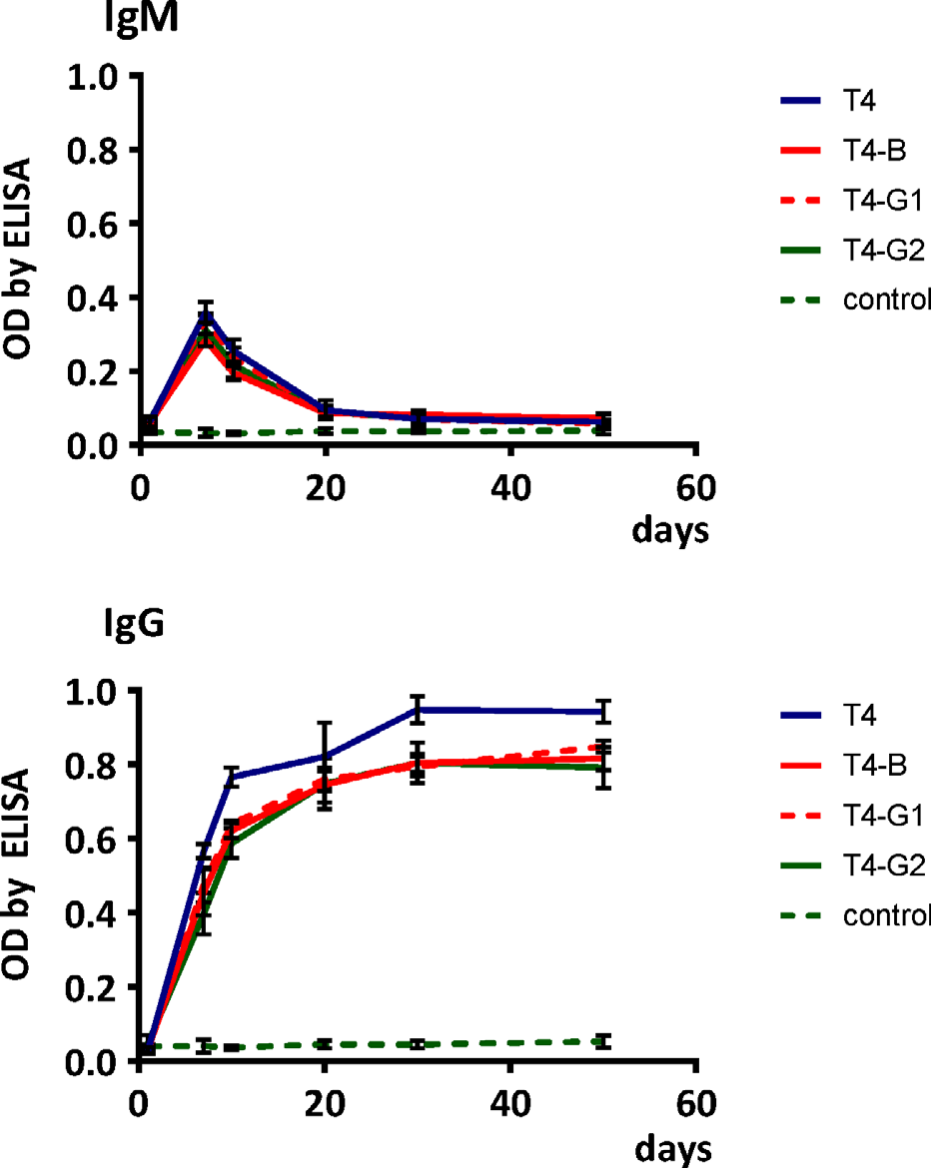
Kinetics of phage‐specific antibody production in mice treated with T4 phages presenting targeting peptides. Upper panel: induction of IgM specific to phages targeting brain (T4‐B) and facilitating translocation from gut to circulation (T4‐G1, T4‐G2), days 1–50 after injection, is presented; lower panel: induction of IgG specific to phages targeting brain (T4‐B) and facilitating translocation from gut to circulation (T4‐G1, T4‐G2). Mice (*N* = 5) were injected with modified phages i.p. at 1 × 10^9 ^pfu per mouse; in each case, non‐modified T4 phage (T4, 1 × 10^9^ pfu per mouse) was used as a control. Blood was collected from tail veins on days 1, 7, 10, 20, 30, 50 and specific antibodies were assessed by ELISA; optical density (OD) by ELISA is presented (points with trend line) with standard deviation (whiskers).

Innate immunity and adaptive immunity are linked by many pathways and they collaboratively contribute to resulting immune responses to foreign antigens. Complement‐conjugated antigens are effective in rapid development of the specific immune response (Dempsey *et al*., 1996). Thus, we investigated whether differences in phage abilities to interact with innate immunity eventually affected the adaptive immunity response, specifically phage‐specific antibody production. However, we did not observe significant differences between levels of specific IgM and IgG elicited in mice in response to treatment with engineered phages T4‐B, T4‐G1, T4‐G2 and unmodified T4. The following typical pattern of induction was observed: the IgM peak was observed around day 7, IgG increased gradually from the beginning of the experiment, a major rise of IgG was noted approximately from day 7, and the IgG level remained high until the end of the experiment on day 50 (Fig. 5). This is in line with antibody induction patterns observed in murine models for other *Myoviridae* bacteriophage F8 (Hodyra‐Stefaniak *et al*., 2015).

## Discussion

**Figure 6:**
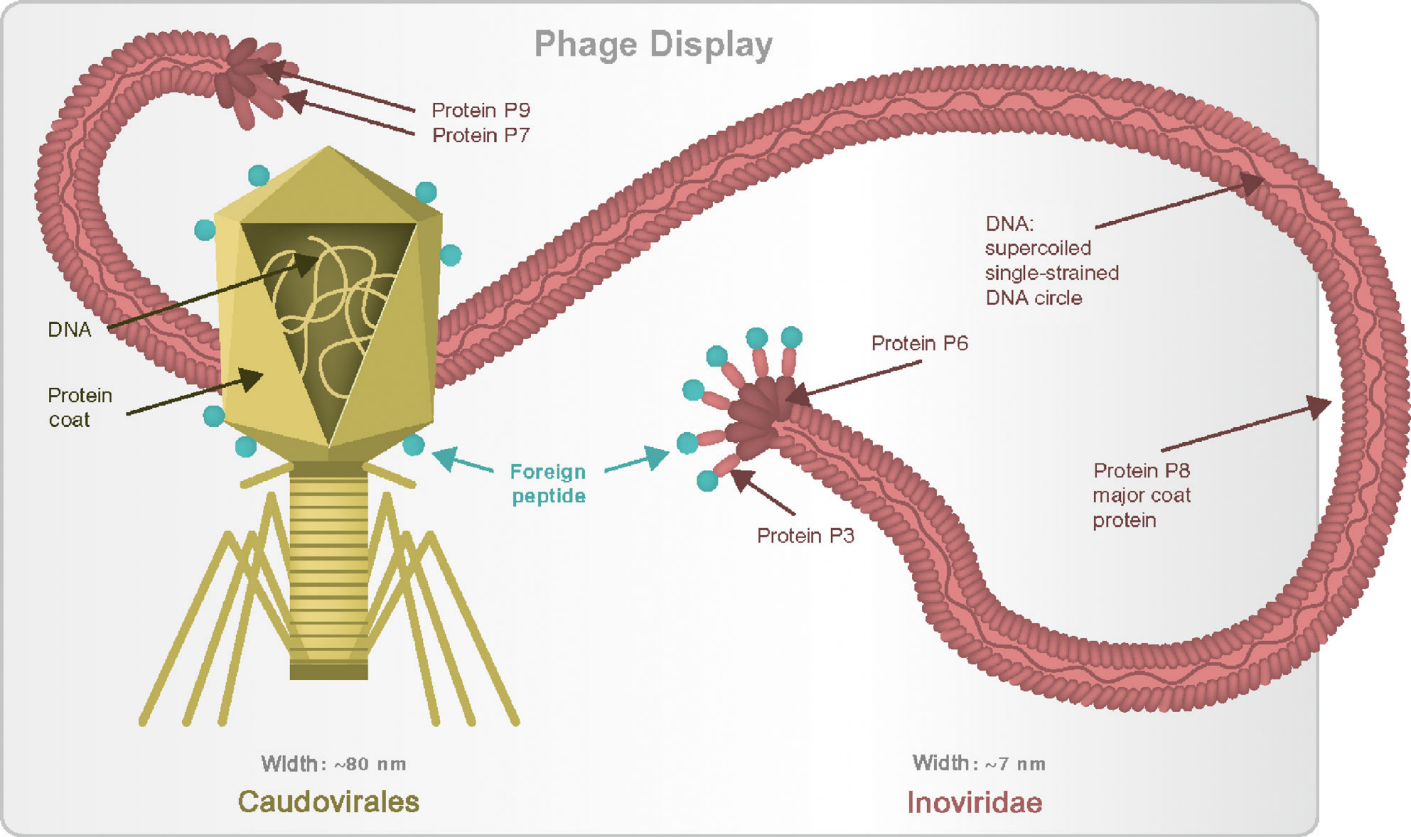
Structural differences between T4 phage‐derived and filamentous phage‐derived vectors presenting tissue targeting peptides.

In this study, we studied engineered bacteriophages presenting tissue‐homing peptides, by evaluating their tissue accumulation and blood circulation *in vivo* in an animal model. Although this study was inspired by very encouraging data from selection of tissue‐homing peptides by phage display (Pasqualini and Ruoslahti, 1996; Arap *et al*., 2002a,b; Duerr *et al*., 2004; Kang *et al*., 2008; Giordano *et al*., 2009; Li *et al*., 2011, 2012; Teesalu *et al*., 2013; Gorski *et al*., 2015), we have not observed the expected concentration of engineered bacteriophages in targeted organs, as a result of any phage modifications tested (Fig. 1). Probably, the major difference between the aforementioned studies that allowed for selection of targeting peptides and the study presented herein is the phage display system that was used. Here, we used T4, which is a tailed phage with a large icosahedral head (*Caudovirales*), while those previous studies applied filamentous phages such as fd, M13 or related phages (*Inoviridae*). *Caudovirales* and *Inoviridae* substantially differ in their morphology and in the way that foreign peptides are exposed on the phage, including peptide‐neighbouring and linking elements (Fig. 6). Thus, these differences are probably important enough to impair the targeting properties of peptides when presented on T4.

In some cases (phage T4‐B and T4‐G2), we did not observe phage accumulation in targeted organs, in fact phage titres were significantly lower than that of unmodified phage. This was opposite to the expected results. However, lower phage titres in targeted tissues correlated with lower titres in spleens and livers (Fig. 1), which suggested that phage titre in the whole body was generally lower. The range of this decrease varied from only 2‐fold up to 100‐fold 2 hours after application. Such a situation may result from impaired phage survival in a living system. Impaired phage survival in the circulation was indeed demonstrated for T4‐B and T4‐G2, when compared to unmodified T4 (Fig. 2). Thus, in addition to *long‐circulating phages* (Merril et al., 1996), we have observed *short‐circulating phages*.

Seeking the mechanisms underlying the *short‐circulating* phenotype, we investigated the immunity reactions to phages. Pharmacokinetics in naïve animals is determined by interactions with innate immunity, where the major roles can be played by phagocytes and possibly by the complement system. We have not found any important effect of phagocytosis (Fig. 4), but the effect of complement on phages was significant. Phage activity decreased after phage exposure to complement by almost 2 orders of magnitude (Fig. 3). Engineered phages T4‐B and T4‐G2 were inactivated by the complement system significantly more strongly than non‐modified T4 (Fig. 3). Although we did not find an important role of phagocytes in the observed phenotype, one should note that in a living system complement and phagocytes cooperate. Foreign objects when opsonized by complement proteins are more readily engulfed by phagocytes. This means that in more natural situation, phagocytosis of these short‐circulating phages can also be more effective, even though the primary reason is their reactivity to the complement system.

Complement proteins (C3b) form covalent bond to surface of targeted object (e.g. a virus) and they recruit further components of the cascade, eventually forming a large structure (with a major contribution of C6, C7, C8 and C9). This complex measures 30.5 nm in its longest dimension, and the upper rim width of 24 nm (Serna *et al*., 2016). This means that each complex, while many can be formed at each phage, have considerable dimensions comparing to phage (approx. head 111 × 78 nm, tail 113 × 18 nm, baseplate 52 × 27 nm). We hypothesize that protein complexes formed by the complement system are at least able to block normal functions of phage proteins, including their steric rearrangements and their adhesion to bacterial surfaces. It is unclear if phage particles can be disrupted by complement system complexes, but natural activity of these complexes is to perforate targeted objects.

Sokoloff *et al*. (2000, 2001, 2004) observed that interactions of T7‐based phage vectors with the complement system were mediated by natural IgM antibodies. Natural IgM antibodies are considered to contribute to important immunoregulatory and housekeeping functions in mammals by recognition of apoptotic cells and enhancing their phagocytic clearance, but their full repertoire of function has not been determined yet (Gronwall *et al*., 2012). In T7 phage display vectors, natural IgM recognized and bound phage particles, eventually activating the complement cascade. Importantly, binding of IgM to phage vectors depended on C‐terminal sequences of peptides presented on capsids. A *long‐circulating* phenotype in rats was generated by presenting peptides with C‐terminal lysine or arginine (Sokoloff *et al*., 2000).

**Figure 7:**
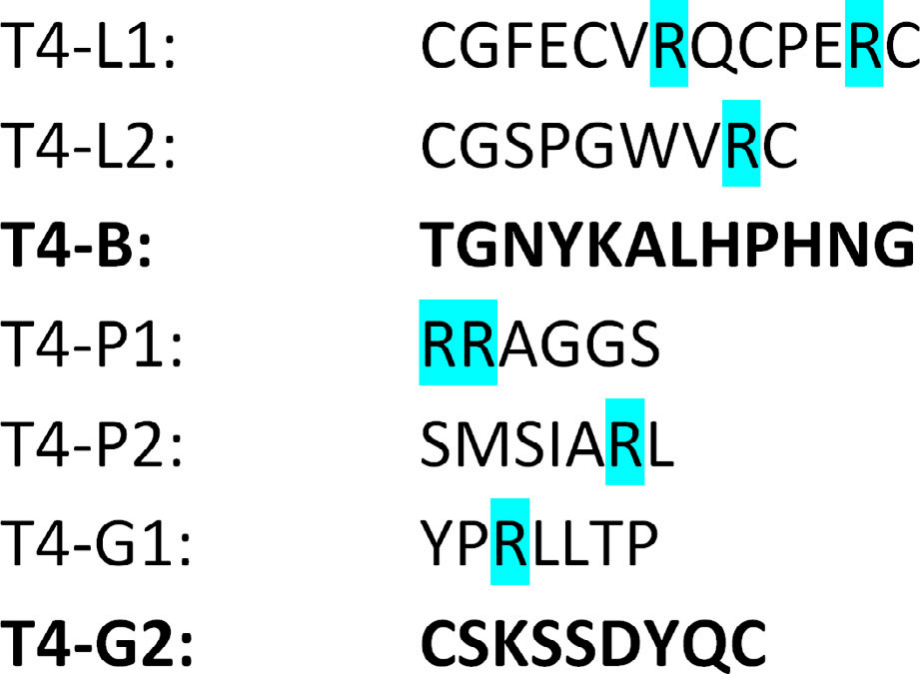
Amino acid sequences of peptides presented on engineered bacteriophages. Arginine (R, blue) was previously identified as attenuating phage interactions with complement system, bold – peptides mediating short‐circulating phenotype of peptide‐presenting bacteriophages.

In our study reported herein, foreign peptides presented on the phage capsid did not expose their C‐terminus, since the fusion to Hoc protein on T4 phage was N‐terminal. However, Vitiello *et al*. (2005) reported that in phage lambda, the *long‐circulating* phenotype (in mice) was mediated by a mutation located inside the gene coding major capsid protein; this mutation resulted in substitution of glutamic acid for lysine inside the relevant protein (not at the C‐terminus) (Vitiello *et al*., 2005). Although the authors proposed lower susceptibility of mutated phage to capture by the mononuclear phagocytic system (referred by them as reticulo‐endothelial system), it is possible that the *long‐circulating* phenotype might instead result from lower phage susceptibility to inactivation by the complement system. Thus, studies of Sokoloff *et al*. (2000) and Vitiello *et al*. (2005) suggest that exposure of lysine (K) or arginine (R) on the phage head surface helps the phage to escape the immune response (*long‐circulating* phenotype), while lack of these two makes the phage more sensitive to inactivation *in vivo*. We have not found exactly the same correlation among the investigated targeting peptides, since lysine (K) was present in two of the peptides mediating the *short‐circulating* phenotype. However, all peptides making T4 phage *short‐circulating* lacked arginine (R), which was present in all others (Fig. 7). This may suggest that the lack of arginine contributes to the *short‐circulating* phenotype. We propose the amino acid composition of phage capsids as the factor strongly affecting phage interaction with the complement system. This is in line with the fact, that an important step of complement action is formation of a covalent bond between C3b complement proteins and surface carbohydrate or protein of targeted object (e.g. a virus). Thus, changes in biochemical characteristics of these surfaces may change vulnerability to complement action. Nevertheless, this mechanism needs further investigation and so far should be regarded as a hypothesis.

One should consider that modifications in the phage particle that make it a phage display platform, not the foreign peptides, may affect phage circulation in a living animal. The engineering itself may affect the phage. In this study, a phage deprived of its surface protein Hoc served as the platform for presentation of the peptides; the phage display system was used to put Hoc fused to the tested peptides on the platform. Thus, differences between the platform and the wild‐type phage may play a role in the observed effects. We also observed differences between relative saturation of phage particles with the fusions, but no correlation between this saturation and impaired phage pharmacokinetics was found. This suggests that the observed results did not derive from differences in the saturation of phage particle with fusions. However, in our opinion, some effect of differences between wild phage particles and those deprived of the surface proteins cannot be fully excluded and they may contribute to the resulting effect of phage engineering on phage pharmacokinetics. The problem should be a subject of further investigations.

The *short‐circulating* phenotype seems unfavourable in therapeutic application of phages. Rapid elimination of phages results in a low overall phage titre in the body, which makes it difficult to achieve the ‘inundation threshold’ that is necessary to control infection (Abedon, 2011). Poor accumulation of phages in tissues is not a good predictor of therapeutic outcomes. Obviously, more studies are needed to understand if and how our results on phage circulation and organ penetration are relevant for clinical phage therapy. Phage penetration may be species‐specific, and other routes of phage administration should also be tested to draw conclusions which may be applicable to treatment of patients. However, changes of phage pharmacokinetics that result from phage engineering should be taken into account in attempts to engineer phages or phage‐derived delivery vectors for medical purposes (e.g. in design of nanocarriers). Designed modifications need to be tested for their effect on phage pharmacokinetics, which may differ between different types of bacteriophages and different types of modifications. Specifically, some coliphages, such as T4, may have developed mechanisms for escaping the immune response in mammals, since mammals are a natural environment for these phages. Coliphages can be easily modified due to the abundance of *Escherichia coli* laboratory strains, coli‐related vectors and well‐established systems for phage display, but also other types of phages may offer a good platform for modifications. Thus, careful selection and individual testing of phage strains designed for particular applications seem to be crucial for their successful use *in vivo*.

## Conclusions

Natural bacteriophages seem to be naturally optimized (to some extent) to circulate in mammalian bodies escaping rapid neutralization, at least those phages that propagate on commensal bacteria of animals and humans. This is in line with evolutionary selection pressure: circulating inside mammalian bodies, phages benefit from evading activity of the immune system. They remain active for longer, thus increasing their chance of encountering sensitive bacteria among mammalian commensals. The complement system contributes to phage neutralization *in vivo*, and thus phages less prone to its activity benefit from longer survival. Phage engineering, especially presenting foreign elements on the phage surface, can destroy optimized biochemical properties of a phage particle and make the phage more visible to the immune system. Eventually, pharmacokinetics of engineered phages differ from that of natural ones, and it can be unfavourable for therapeutic use: the phage may acquire the *short‐circulating* phenotype. However, even when more prone to innate immunity action, short‐circulating phages do not induce a stronger specific immune response. Unfavourable effects of phage engineering can manifest in one type of bacteriophage (e.g. *Caudovirales*), while not being observed in others (e.g. filamentous phages). Thus, careful selection and individual testing of phage strains designed for particular applications seem to be crucial for their optimal use *in vivo*.

## Experimental procedure

### Bacteriophages

The wild‐type T4 phage was purchased from American Type Culture Collection (ATCC) (Rockville, Maryland, USA). Phage display of targeting peptides on T4 was completed as previously described with minor modifications (Oslizlo *et al*., 2011). Briefly, a phage variant without the protein Hoc was cultured on *E. coli* transformed with expression vectors coding Hoc‐targeting peptide fusions (N‐terminal), that is, fusions were incorporated into phage capsid during natural phage assembly inside bacteria. Expression vectors were constructed in pCDFDuet‐1 (Novagen) by cloning of PCR products coding Hoc‐targeting peptide fusions into the XhoI/BglIII restriction site. The sequences coding for targeting peptides were introduced in a PCR reaction from the forward primer. Peptides fused to Hoc and displayed on T4 phage are listed in Table 1. All cloning vectors were directly sequenced for control of construction accuracy and tested for effective expression of the Hoc fusions in *E. coli* B834 (OverExpress). Appropriate clones were used for the phage display cultures.

Lysates were purified by filtration through polysulfone membranes and by chromatography: gel filtration on Sepharose 4B (Sigma‐Aldrich, Poland). The preparation was dialysed using 1000 kDa‐pore membranes against sterile and pure PBS and filtered with 0.22 μm PVDF filters (Millipore, Europe). Phage concentrations were measured by the double‐layer method of Adams or by routine test dilution (RTD), and presence of Hoc fusions was confirmed by ELISA with anti‐Hoc murine serum (Supporting Information) (Dabrowska *et al*., 2014a). LPS concentration was controlled by EndoLISA (Hyglos GmbH, Germany), according to the manufacturer's instructions. Diluted samples or standard dilution with binding buffer were incubated overnight at room temperature with shaking. Subsequently, the plate was washed and assay reagent was added. The fluorescent signal was detected immediately in a fluorescence reader (Synergy H4 H4MLFPTAD BioTek Instruments USA). Effective concentration of LPS was less than 1 EU per mouse or per ml of solutions used for *ex vivo* complement and phagocytosis assays.

### Animal model of phage circulation in vivo

The male BALB/c (6–10 weeks) mice were purchased from the Center of Experimental Medicine, Medical University of Bialystok, Poland, or Mossakowski Medical Research Centre, Polish Academy of Sciences, Warsaw, Poland and bred under specific pathogen‐free (SPF) conditions in the Animal Breeding Center of the Institute of Immunology and Experimental Therapy (IIET).

For the study of engineered phages in targeted organs, spleen and liver, mice (*N* = 5 to 6) were (i) injected intraperitoneally with 5 × 10^9^ pfu per mouse of phages targeting lungs, brain or prostate (T4‐L1, T4‐L2, T4‐P1, T4‐P2, T4‐B), phage titre being measured in the tissues 2 h later, or (ii) treated with phage added to drinking water 5 × 10^10^ pfu per ml of phages modified with peptides facilitating translocation from gut to circulation (T4‐G1, T4‐G2), and phage titre was measured 4 h later in blood. In each case, non‐modified T4 phage that was cultured and purified identically to modified phages was used as a control. Animals were sacrificed by cervical dislocation, liver and spleen were excised in all animals, and targeted organs were excised according to the tested phage type. Organs were homogenized and weighed, and the homogenates were serially diluted with PBS (1 g equalling 1 ml). The phage titre in each tissue/organ was determined by the RTD method. In the case of phages modified with peptides facilitating translocation from gut to circulation, murine blood was collected from the tail vein into heparinized tubes, under local anaesthesia (lidocaine) and phage titre was determined by the RTD method.

For the study of engineered phages’ survival in circulation, mice (*N* = 5 to 6) were injected intravenously 1 × 10^9^–1 × 10^10^ pfu per mouse and their titre in blood was measured 2 hours later. In each case, non‐modified T4 phage that was cultured and purified identically to modified phages was used as a control. Blood was collected from the orbital plexus vein into heparinized tubes, under anaesthesia, and phage titre was determined by the RTD method.

### Animal model of antibody induction

The male C57Bl6/J (6–10 weeks) mice were purchased from the Center of Experimental Medicine, Medical University of Bialystok, Poland, and bred under SPF conditions in the Animal Breeding Center of the IIET.

Mice (*N* = 5 to 6) were injected intraperitoneally (i.p.) with modified phages T4‐B, T4‐G1 or T4‐G2; the dose was 1 × 10^10^ pfu per mouse. Non‐modified T4 phage that was cultured and purified identically to modified phages was used as a control. Blood was collected from tail veins on days 1, 7, 10, 20, 30 and 50; blood was collected into clotting tubes. Serum was separated from the blood by double centrifugation at 2250 × *g* and used for the ELISA assay.

ELISA was conducted on MaxiSorp flat‐bottom 96‐well plates (Nunc, Thermo Scientific, Europe) that were covered overnight with phages (5 × 10^9^ pfu ml^−1^). Subsequently, wells were washed with PBS and blocked with 1% SuperBlock Blocking Buffer (Thermo Scientific, Europe). Diluted serum (1/100 in PBS) was added (100 μl of diluted serum per well). The plate was incubated at 37°C for 2 h and washed with 0.05% Tween 20 in PBS (Serva, Europe) five times. Diluted detection secondary antibody was applied (100 μl per well): peroxidase‐conjugated AffiniPure goat anti‐mouse IgM (Jackson ImmunoResearch Laboratories) or peroxidase‐conjugated AffiniPure goat anti‐mouse IgG (Jackson ImmunoResearch Laboratories). The plate was incubated for 1 h at room temperature in the dark. TMB substrate reagents for peroxidase were used according to the manufacturer's instructions (DY999, R&D Systems, Europe) and incubated for 20 min. Twenty‐five μl of 2N H_2_SO_4_ was added, and absorbance was measured at 450 nm (main reading) and 570 nm (background).

### Phage inactivation by the complement system

The human complement effect on engineered bacteriophages was evaluated in sera of 8 healthy volunteers, with their written consent, and in accordance with the local Commission of Bioethics, Wroclaw Medical University (approval no. 503/2015). Potential deficiencies of the complement activity in the donors were tested by the diagnostic Complement System Screen (WIESLAB, Euro Diagnostica AB, Sweden) before the experiment, and samples with any abnormal complement system activity were excluded. Blood was collected into clotting tubes, and serum was separated from the blood by double centrifugation at 2250 g. Half of each serum sample was heat‐inactivated by incubation at 56°C for 1 h to inhibit the serum complement system. Each donor's serum was further tested as a complement‐inactivated or non‐inactivated serum. To test the inhibitory effect of human sera on bacteriophages, each engineered bacteriophage – T4‐L1, T4‐L2, T4‐P1, T4‐P2, T4‐B, T4‐G1, T4‐G2 (10^7^ pfu ml^−1^) – and T4 as the control (10^7^ pfu ml^−1^) was mixed with serum samples (1:1) and incubated at 37°C for 1 h. After incubation, phage activity was tested by the double‐layer plate method.

### Phage inactivation by phagocytes

Human phagocytes’ effect on engineered bacteriophages was evaluated in sera of 6 healthy volunteers, with their written consent, and in accordance with the local Commission of Bioethics, Wroclaw Medical University (approval no. 503/2015). Fractionation and isolation of human peripheral blood phagocytes from heparinized blood samples (polymorphonuclear neutrophils (PMNs) and mononuclear blood cells (PBMCs)) were conducted according to Boyum (Boyum, 1968) with modifications. Briefly, PBMCs and PMNs were isolated using a density gradient (Histopaque 1119 and Histopaque 1077 (Sigma‐Aldrich)) by centrifugation (700*g*, 30 min, 20°C). The PMN‐rich layer of Histopaque 1119 and mononuclear‐cell‐rich layer of Histopaque 1077 were collected, and the cells were washed (5 min, 870 × *g*, 4°C) in PBS three times. Morphology, quantity and viability of the cells were verified by optical microscopy in the histological Bürker chamber. The cells were then diluted in Hanks solution supplemented with 5% fetal calf serum (FCS) to achieve the required cell density for the further experiments.

Phagocytosis was tested in 24‐well plates. Cells were used in a final density of 10^6^ cells per ml. Phages T4‐B1, T4‐G1, T4‐G2 and T4 as the control were added to a final count of 10^5^/ml (volume: 1 ml). Cultures were incubated for 1.5 h at 37°C, in 5% CO_2_ with occasional gentle mixing. Samples were centrifuged (200 × *g*, 4°C, 5 min) and viable phage titre was determined by RTD in the culture supernatant.

### Ethics statements

All experiments with human samples were approved by the local Commission of Bioethics, Wroclaw Medical University (no. 503/2015), and they were conducted only with the written consent of a healthy volunteer who entered the study as a blood donor.

All animal experiments were performed according to EU Directive 2010/63/EU for animal experimentations and were approved by the 1st Local Committee for Experiments with the Use of Laboratory Animals, Wroclaw, Poland (no. 07/2017 and no. 71/2015). The authors followed the ARRIVE (Animal Research: Reporting of In Vivo Experiments) guidelines (Kilkenny *et al*., 2012).

### Statistics

All experiments were repeated 2–3 times; they were not summarized; exemplary experiments with their individual N values and statistical significance were presented. Statistical analysis was performed using the Kruskal–Wallis ANOVA or Mann–Whitney *U*‐test with the Statistica 8.0 software package (www.statsoft.pl).

## Conflict of interest

None declared.

## Supporting information


**Fig. S1.** Saturation of phage particles with Hoc‐peptide fusions.

**Fig. S2.** Comparison of engineered phages and T4 phage affinity to targeted cells.
