# Supplementary figures and images for: Bacteriophages engineered to display foreign peptides may become short‐circulating phages
Source: Microb Biotechnol. 2019 Apr 29;12(4):730–41. doi: 10.1111/1751-7915.13414 (PMC6559017; doi:10.1111/1751-7915.13414)

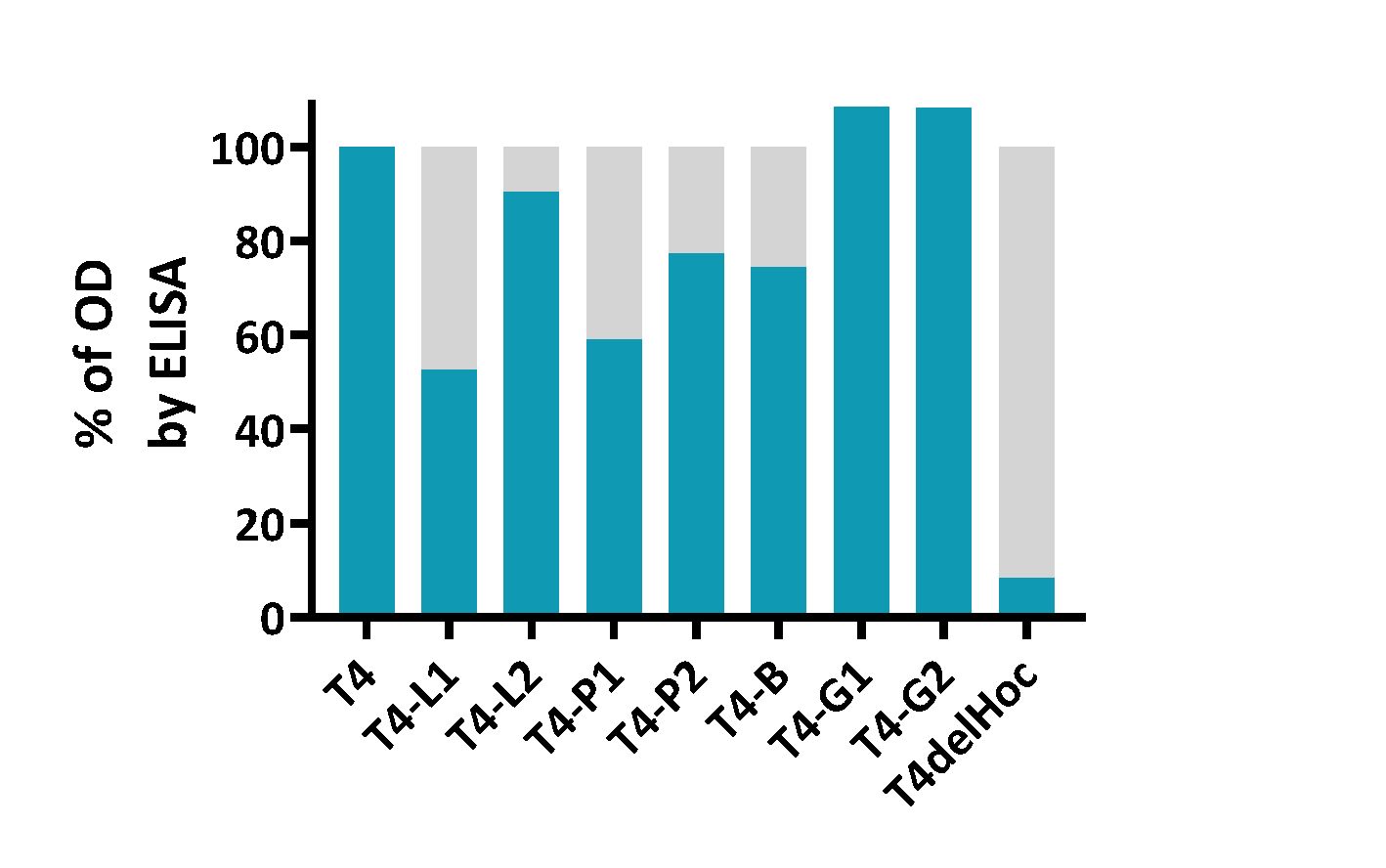

Supplement: Supplementary file 1 — Fig. S1. Saturation of phage particles with Hoc‐peptide fusions. [file MBT2-12-730-s001.tiff]

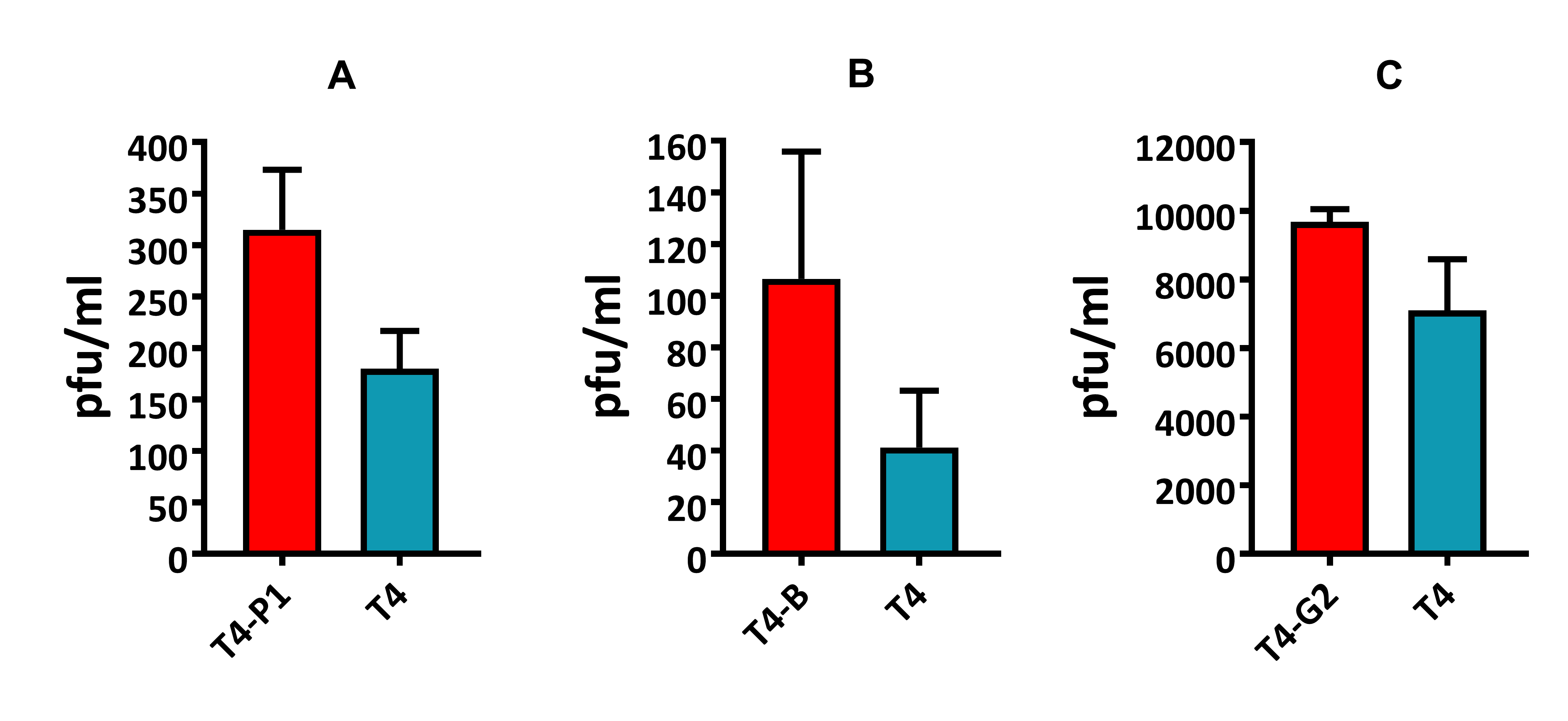

Supplement: Supplementary file 2 — Fig. S2. Comparison of engineered phages and T4 phage affinity to targeted cells. [file MBT2-12-730-s002.tif]
