# Supplementary material for: Bacteriophages engineered to display foreign peptides may become short‐circulating phages
Source: Microb Biotechnol. 2019 Apr 29;12(4):730–41. doi: 10.1111/1751-7915.13414 (PMC6559017; doi:10.1111/1751-7915.13414)
Supplement: Supplementary file 3 [file MBT2-12-730-s003.docx]

Figure S1. Saturation of phage particles with Hoc-peptide fusions. Hoc detection was completed by ELISA with murine Hoc-specific serum. Engineered phages: T4-L1, T4-L2, T4-P1, T4-P2, T4-B, T4-G1, T4-G2, as described in the main manuscript. T4- positive control where detected OD by ELISA was denoted as 100% (wild type phage with natural Hoc proteins), T4delHoc- negative control (T4 phage mutant without protein Hoc on its capsid). Blue bars- relative saturation of phage particles with Hoc proteins as detected OD by ELISA recalculated to % of positive control.

Figure S2. Comparison of engineered phages and T4 phage affinity to targeted cells; Panel A: phage binding to LnCAP (prostate) cells after 30 min incubation in 37ºC, cells were washed 2 times with PBS, phage concentration in cell pellet resuspended in 200 μl PBS is presented; Panel B: phage binding to bENd.3 (brain) cells after 30 min incubation in 37ºC, cells were washed 2 times with PBS, phage concentration in cell pellet resuspended in 200 μl PBS is presented; Panel C: phage transcytosis across Caco-2 cell monolayer 2 hours in 37ºC, according to Nguyen et al. (2017), phage concentration in the lower chamber is presented.
